# Expression level of EGFR and MET receptors regulates invasiveness of melanoma cells

**DOI:** 10.1111/jcmm.14730

**Published:** 2019-10-22

**Authors:** Katarzyna Pietraszek‐Gremplewicz, Aleksandra Simiczyjew, Ewelina Dratkiewicz, Marta Podgórska, Ilona Styczeń, Rafał Matkowski, Marcin Ziętek, Dorota Nowak

**Affiliations:** ^1^ Department of Cell Pathology Faculty of Biotechnology University of Wroclaw Wroclaw Poland; ^2^ Department of Oncology and Division of Surgical Oncology Wroclaw Medical University Wroclaw Poland; ^3^ Lower Silesian Oncology Center Wroclaw Poland

**Keywords:** EGFR, invadopodia, invasion, melanoma, MET

## Abstract

Epidermal and hepatocyte growth factors can stimulate invasive abilities of melanoma cells, while treatment with combination of their receptors' (EGFR and MET, respectively) inhibitors reduces viability of these cells, as we have previously shown. Proposed therapy has potential; however, used drugs block more than one goal effectively, what raises the question about the real target of analysed inhibitors. For this reason, we analysed direct involvement of these receptors in the invasion of melanoma cells inducing EGFR and MET up‐ and down‐regulations in examined cells. Results were acquired with assays evaluating cell migration and invasion (scratch wound assay, Transwell filter‐based method and single‐cell tracking). We revealed that cells' motile abilities are increased after EGFR overexpression and decreased following EGFR and MET silencing. This outcome correlates with elevated (EGFR up‐regulation) or reduced (EGFR/MET down‐regulation) number of formed invadopodia, visualized with immunofluorescence, and their rate of proteolytic abilities, evaluated by fluorescent gelatin degradation assay, and gelatin zymography, compared to control cells. Above‐mentioned data indicate that both—EGFR and MET signalling is directly connected with melanoma cells invasion, what establishes these receptors as promising targets for anti‐cancer treatment.

## 1 INTRODUCTION

Melanoma is a heterogeneous tumour with a very low cure rate in the case of metastasis in which conventional therapies fail to improve overall survival. Although many genes important for melanoma induction, transformation and metastasis have been identified, the established targeted therapies are often inefficient in the final outcome. This phenomenon may be related to the incomplete knowledge of the process of melanoma progression including possible mechanisms leading to development of drug resistance. Understanding the acquisition of invasive behaviour by melanoma cells is therefore crucial. The research focused on the molecules and pathways involved in its progression is also needed.^1^

Metastasis, the main cause of cancer patients' mortality, is a multi‐step process, where cancer cells spread from primary tumour into the distant tissues moving through the surrounding extracellular matrix (ECM). Cell invasion is an essential stage of cancer spreading involving ECM degradation and remodelling.^2^ In recent years, actin‐rich protrusions known as invadopodia have been shown to be critical for migration through the ECM.^3^ These structures consist of an actin core surrounded by a number of protein components, including cytoskeletal modulators, adhesion proteins, scaffolding proteins and signalling molecules. Their main role is secretion of proteases digesting elements of the ECM, what enables cancer cells to migrate through surrounding microenvironment.^4^, ^5^ Previously, we showed that chemoattractants—epidermal growth factor (EGF) and hepatocyte growth factor (HGF) stimulate invadopodia formation, and extracellular matrix degradation, what correlates with higher invasive abilities of melanoma cells.^6^

EGF receptor (EGFR) is up‐regulated in many types of cancer. In the case of melanoma, the gene expression data are inconsistent^7^; however, some researches postulate that overexpression of *EGFR* often occurs in advanced stages of melanoma.^8^ Following ligand binding EGFR undergoes dimerization what induces its autophosphorylation and is essential for downstream signalling pathways activation, with the most significant represented by PI3K/AKT (Phosphoinositide 3‐kinase/Protein kinase B) and MAPK (mitogen‐activated protein kinase). These cascades participate in the regulation of several cellular processes, including cell proliferation, prevention of apoptosis and promotion of cell invasion.^9^ Therefore, any aberrations in EGFR expression level or activity might be linked to the higher ability of cancer cells to invade and form metastasis.^10^

The level of HGF receptor (MET) also seems to be related to the stage of malignancy in melanoma.^11^ Its activation, mediated by HGF binding, promotes several processes involved in oncogenesis including tumour cell proliferation, migration, invasion and metastasis, through several intracellular signalling pathways such as PI3K/AKT, Src, STAT3 (Signal transducer and activator of transcription) and MAPK.^12^ Moreover, MET localizes to invadopodia along with cortactin, one of the main components of migratory protrusions, and regulates its activation.^13^ Interestingly, it was shown that both—EGFR and MET signalling regulates invadopodia formation and degradation of ECM by breast cancer cells.^13^, ^14^

Both receptors—EGFR and MET seem to be a promising target in anti‐metastatic therapy, since our previous studies indicated that chemical inhibition of their activity results in synergistic cytotoxic effect on the viability and proliferation of melanoma cell lines derived from primary tumour and metastasis.^15^ Additionally, we observed the reduction in number of formed invadopodia and decline of migration, and invasion capacity of breast cancer cells treated with EGFR, and MET inhibitors.^16^ Despite the fact that use of chemical inhibitors appears to be a good strategy in the anti‐melanoma therapy, there appears to be a problem with low specificity of these compounds which may block activity of several receptors. This in turn may generate some ambiguities related to the targets, against which the therapies should be directed. Therefore, in this work we focused on the analysis of direct involvement of EGFR or MET in the regulation of invasiveness of melanoma cells derived from primary tumour and metastasis.

## 2 MATERIALS AND METHODS

### 2.1 Cell culture

The human melanoma A375 (primary) cell line was obtained from the American Type Culture Collection (ATCC), whereas WM9 (metastatic) cell line was obtained from Rockland Immunochemicals, Inc. Cells were grown in tissue culture flasks (Eppendorf) at 37°C in 5% CO_2_/95% humidified air in DMEM medium with lowered NaHCO_3_ (1.5 g/L) (IITD PAN, Wrocław, Poland) containing 10% FBS, 2 mmol/L glutamine and antibiotics (100 U/mL penicillin, 100 μg/mL streptomycin) (Invitrogen) and passaged using 0.25% trypsin/0.05% EDTA solution (IITD PAN, Wrocław, Poland) twice a week.

### 2.2 Transfection procedure

Cells were transfected with 29‐mer shRNA constructs directed against human *EGFR* or *MET* or 29‐mer non‐targeting shRNA (shCTRL), which were purchased from OriGene. For EGFR overexpression, pcDNA3 plasmid (Invitrogen) with cloned cDNA encoding human *EGFR* was applied, and cells transfected with the empty pcDNA3 plasmid (MOCK) constituted control cells. Lipofectamine 3000 (Invitrogen) was used to transfect the cells according to the manufacturer's protocol. Transfected cells were purified by at least 2 weeks selection based on puromycin (0.5 µg/mL) (Santa Cruz Biotechnologies) or G418 (1 mg/mL) (Santa Cruz Biotechnologies) antibiotics for shRNA or pcDNA3 constructs, respectively. Expression of *EGFR* and *MET* in all obtained cells was monitored by real‐time PCR and Western blotting methods.

### 2.3 qRT‐PCR analysis of gene expression

To measure the expression level of *EGFR* and *MET* in obtained cell lines, total RNA was isolated using GenElute™ Mammalian Total RNA Miniprep Kit (Sigma‐Aldrich) following the manufacturer's protocol. After DNase I (Sigma‐Aldrich) treatment, reverse transcription reaction was performed using 0.5 μg of RNA and the High Capacity cDNA Reverse Transcription Kit (Applied Biosystems) following the manufacturer's instructions. Quantitative PCR was performed using StepOne Plus Real‐Time PCR (Applied Biosystems) in a mixture containing TaqMan^®^ Universal Master Mix II (Applied Biosystems), 10 ng of cDNA and specific probes in a total volume of 10 μL. The following TaqMan^®^ probes were used: GAPDH (Hs02758991‐g1), EGFR (Hs01076091‐m1) and MET (Hs01565576‐m1), (Applied Biosystems). GAPDH (glyceraldehyde 3‐phosphate dehydrogenase) served as a housekeeping gene. Relative quantification of gene expression was calculated based on the comparative C_T_ (threshold cycle value) method (ΔC_T_ = C_T__gene_ _of_ _interest_ − C_T__housekeeping_ _gene_). Three independent experiments were performed for all cell lines.

### 2.4 Western blotting analysis

To detect the protein level of EGFR and MET, cell lysates were prepared from examined cells by harvesting them in urea buffer (50 mmol/L TRIS‐HCl pH 7.4, 5% SDS, 8.6% sucrose, 1 mmol/L DTT, 0.45% urea), supplemented with protease inhibitors cocktail (Sigma‐Aldrich). Protein concentration was determined by the Bradford procedure,^17^ and an identical amount of proteins were separated by SDS‐PAGE electrophoresis^18^ and transferred to nitrocellulose sheets.^19^ Then, membranes were incubated with suitable primary antibodies directed against EGFR, MET or GAPDH (Santa Cruz Biotechnologies). Next, goat anti‐mouse or goat anti‐rabbit antibodies conjugated with horseradish peroxidase (Cell Signaling Technologies) were applied. Immunoblots were developed using the Clarity Western ECL Substrate (Bio‐Rad), scanned with ChemiDoc (Bio‐Rad) and analysed with ImageLab software (ver. 6.0, Bio‐Rad). At least three independent experiments were performed in each case.

### 2.5 Time‐lapse migration assay

Cells were seeded on 1 mg/mL Matrigel‐coated (Corning) 96‐well ImageLock plates (Essenbioscience). After 24 hours, when the cells reached confluency, standardized wounds were made in all wells simultaneously using Wound Maker™ (Essenbioscience). Phase‐contrast time‐lapse photos were captured using IncuCyte^®^ Live‐Cell Analysis System for 48 hours with a time interval of 2 hours using a 10× objective. An IncuCyte^®^ Scratch Wound Cell Migration Software Module was used for data analysis, and the calculation of relative would density was based on the increase in the area covered by the cells in time. The experiments were performed in triplicate, each condition consisting of four replicates.

For the evaluation of migration distances and cell trajectories, cells were seeded in low density, and images were analysed using ImageJ software with Manual Tracking plugin.^20^ The distance covered by every cell was measured as the total distance based on the cumulative track lengths. The experiments were performed three times, and each time 40 cells were analysed.

### 2.6 Transwell migration and invasion assay

Cell migration and invasion tests were performed using Transwell filters with 8 µm pore size (BD Biosciences) placed in 24‐well plates. Prior to the experiment, cells were starved for 16 hours in serum‐free DMEM medium. Cells were seeded in medium deprived of FBS directly onto Transwell filters (for migration assay) or on filters coated with Matrigel (1 mg/mL) (for invasion assay). At the bottom of the well, medium containing 20% foetal bovine serum was present as a chemoattractant. After 24 hours, the non‐invading cells present on the upper side of the filters were removed. Cells which invaded through the membrane were fixed with 4% formaldehyde, nuclei were stained with Hoechst 33342 (Invitrogen) and counted under the fluorescent microscope (Olympus IX70). The results are presented as a number of the treated cells that migrated through the filter compared with the amount of migrating control cells (relative invasion factor). The experiments were performed three times, and each independent experiment consisted of three measurements.

### 2.7 Fluorescent staining

The subcellular distribution of actin filaments and cortactin was examined by immunofluorescence in cells seeded on 1 mg/mL Matrigel‐coated coverslips in 24‐well plates. After 24 hours, cells were fixed with 4% formaldehyde and permeabilized with 0.1% Triton X‐100 in PBS. Coverslips were then blocked with 1% bovine serum albumin in PBS. Anti‐cortactin antibodies (Santa Cruz Biotechnologies), followed by Alexa Fluor 488‐conjugated anti‐rabbit secondary antibodies (Invitrogen), were applied to visualize this protein. Actin filaments were stained with Alexa Fluor 568‐labelled phalloidin (Invitrogen) and cell nuclei with Hoechst 33 342. Stained cells were visualized using confocal laser scanning microscope, Leica SP8, with LasX 3.3.0 software (Leica, Wetzlar, Germany), and representative pictures of cells are shown. Quantitative analysis of the number of invadopodia per cell was performed using ImageJ software.^20^ Only invadopodia positive for F‐actin and cortactin were scored, and at least 40 cells were analysed per condition.

### 2.8 Fluorescent gelatin degradation assay

The experiments were done as previously described.^21^ Briefly, sterile coverslips coated with poly‐l‐lysine (BD Biosciences) were washed with PBS and incubated with 0.5% glutaraldehyde for 15 minutes at room temperature. Coverslips were washed with PBS and coated with FITC‐conjugated gelatin (Invitrogen) for 10 minutes. After washing with PBS, coverslips were incubated with sodium borohydride for 1 minute and washed with PBS. Cells were seeded in 24‐well plates containing prepared coverslips coated with fluorescent gelatin and incubated at 37°C for 12 hours. Next, cells were fixed with 4% formaldehyde and labelled for filamentous actin with Alexa Fluor 568‐phalloidin. Images were taken using the Olympus FV500 confocal laser scanning microscope and FluoView software (Olympus). Sites of degraded matrix were visible as dark areas (spots) in the bright green fluorescent gelatin matrix. The area of gelatin digestion was calculated for 40 cells per condition using ImageJ software.^20^

### 2.9 Gelatin zymography

The MMP‐9 activity was determined in serum‐free media collected after 48 hours of incubation with cells and concentrated about 20 times using Amicon^®^ Ultra‐4 centrifugal filters (Merck Millipore). Then, after determination of protein concentration by Bradford method,^17^ cell‐conditioned media were analysed on SDS‐polyacrylamide gels containing 1 mg/mL gelatin. Obtained gels were stained with Coomassie Brilliant Blue G‐250 (Sigma‐Aldrich), and MMPs activity was detected as transparent bands present on the blue background. At least three independent experiments were performed.

### 2.10 Statistical analysis

All data are presented as mean ± standard deviation (SD), and their significance was determined using Student's *t* test. The significance test was set at *P *≤ .05 (*), *P *≤ .01 (**) or *P *≤ .001 (***).

## 3 RESULTS

### 3.1 Characterization of the generated cell lines

**Figure 1:**
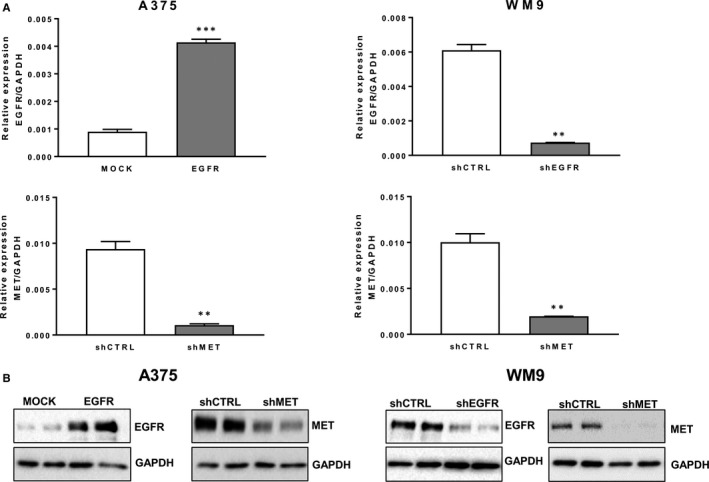
Expression level of EGFR and MET in generated variants of A375 and WM9 melanoma cell lines. A, Results of qRT‐PCR analysis of *EGFR* and *MET* expression are shown as the mean (relative expression compared to GAPDH) ± SD of three independent experiments. (**) *P* ≤ .01, (***) *P* ≤ .001. B, Western blotting analysis of EGFR and MET protein level in generated cell lines. Membranes were probed with antibodies directed against total EGFR and MET, and are representative for at least three independent experiments. GAPDH was used as the sample loading control

In our studies, we used two melanoma cell lines: one isolated from primary amelanotic tumour (A375) and the second derived from lymph node metastasis (WM9). Previously, we demonstrated that both cell lines express HGF receptor at high level, whereas they differ in EGFR expression, which was detected at lower level in A375 in comparison with WM9 cells.^15^ Therefore, to test the influence of MET signalling on the invasive abilities of melanoma cells we decided to generate cell lines with lowered expression level of this protein using shRNA‐based method. Stable down‐regulation of HGF receptor expression in the obtained A375 shMET and WM9 shMET cells in comparison with cells transfected with non‐targeting shRNA (A375 shCTRL, and WM9 shCTRL, respectively) was confirmed at mRNA and protein level (Figure 1A,B).

To analyse the role of EGRF in regulation of invasive abilities of melanoma cells, we generated variant of A375 cell line with stably up‐regulated expression of EGFR (A375 EGFR), in comparison with cells transfected with empty plasmid A375 MOCK (Figure 1A,B). Additionally, we decreased the expression level of EGFR in WM9 cells using shRNA approach, thus generating WM9 shEGFR cell line, what was confirmed by qRT‐PCR and Western blotting analysis (Figure 1A,B).

### 3.2 The level of EGFR and MET regulates migration and invasion abilities of melanoma cells

**Figure 2:**
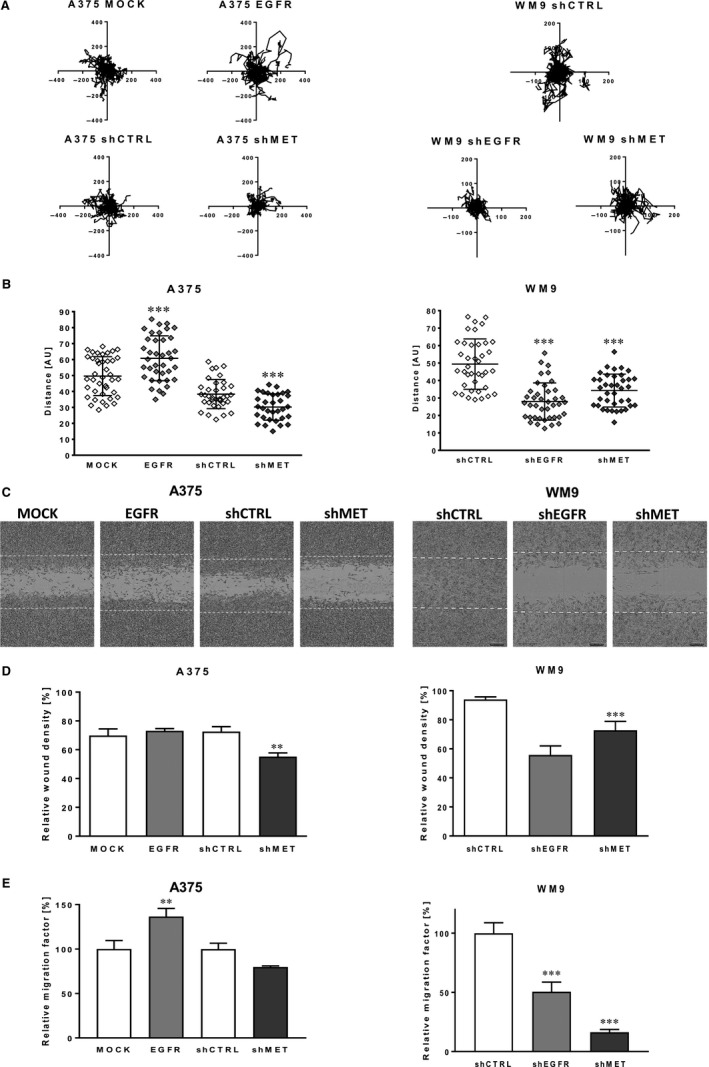
Migration abilities of melanoma cells with altered level of EGFR and MET. A, Cell trajectories and (B) migration distances of single A375 and WM9 cells analysed for 48 h using IncuCyte^®^ Live‐Cell Analysis System and ImageJ software. C, Representative images of wound closure, which was (D) quantified as per cent of area colonized by cells within 48 h (based on pictures analysed with an IncuCyte^®^ Scratch Wound Cell Migration Software Module). E, The migration assay executed on transwell filters for 24 h. Relative migration factor was calculated versus control cells, where number of migrating control cells is set as 100%. Results are expressed as the mean ± SD of three independent experiments. (**) *P* ≤ .01, (***) *P* ≤ .001

Firstly, the influence of EGFR and MET expression level on spontaneous migration, where cells were seeded sparsely, and there was no factor inducing directional migration, was verified (Figure 2A,B). We noticed that A375 EGFR cells were able to cover much longer distances than control A375 MOCK cells. The opposite result was observed in the case of decreased level of EGFR, where WM9 shEGFR cells reached much shorter distances than WM9 shCTRL cells. In the case of both cell lines with silenced MET expression, we obtained similar effects—the A375 shMET and WM9 shMET covered shorter distances in comparison with control. Next, migration imitating movement of cells in two‐dimensional (2D) conditions, for example on the surface of basement membrane, was analysed in directional migration scratch assay (Figure 2C,D). Results of this assay were analogous to these obtained during spontaneous migration assay. Down‐regulation of MET expression led to decreased migration abilities of A375 and WM9 cells. Then, Boyden chamber migration assays were performed (Figure 2E), in which cell migration through Transwell filters was stimulated by the gradient presence of the chemoattractant (FBS). A significant increase in the migration capacity was observed in the case of A375 EGFR cells, while opposite effect was detected in WM9 cells with decreased expression of this receptor. In both cell lines, down‐regulation of MET reduced cell movement; however in the case of A375 shMET cells, this result was not statistically significant.

**Figure 3:**
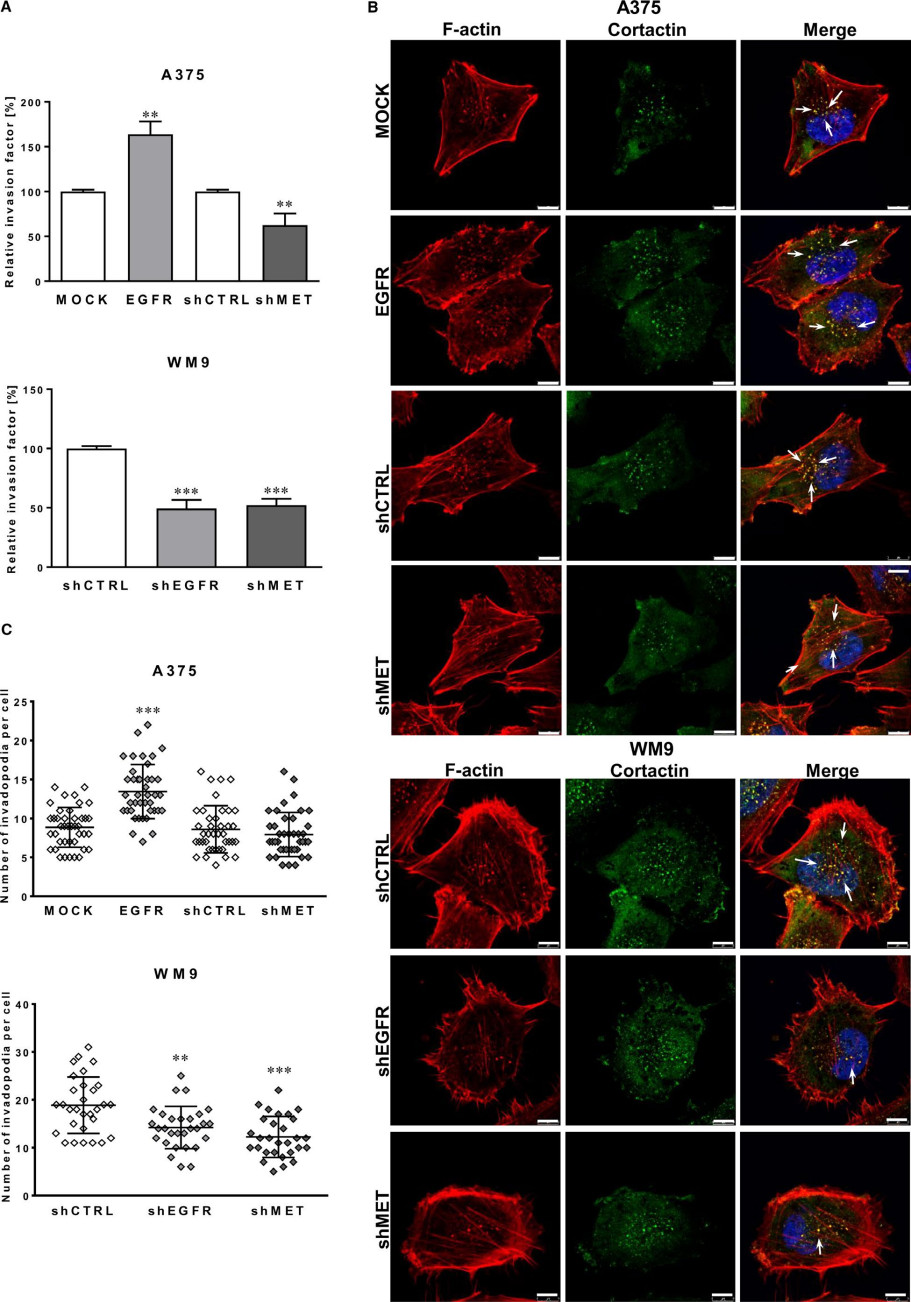
Impact of EGFR and MET on invasion abilities and invadopodia formation in examined melanoma cell lines. A, The invasion assay performed on transwell filters coated with Matrigel for 24 h. Relative invasion factor was calculated versus control cells, where number of invading control cells is set as 100%. Results are expressed as the mean ± SD of three independent experiments. B, Representative pictures of A375 (MOCK, EGFR, shCTRL and shMET) and WM9 (shCTRL, shEGFR and shMET) cells seeded on Matrigel‐coated coverslips stained for F‐actin (red), cortactin (green) and cell nuclei (blue). Arrows indicate invadopodia. Scale bar—8 μm. C, Quantification of the average number of invadopodia in examined cells. Invadopodia formed by at least 40 cells from three independent experiments were counted, and results are presented as the mean ± SD. (**) *P* ≤ .01, (***) *P* ≤ .001

To analyse whether modified protein levels of MET and EGFR are able to impact melanoma cell migration in three‐dimensional (3D) conditions, the invasion assays were subsequently performed (Figure 3A). We observed that tested cells invade through Matrigel layer in a similar way as they migrate in 2D conditions. Overexpression of EGFR stimulated the invasion of A375 EGFR cells, whereas in the case of silenced EGFR and MET expression decreased invasion capacity was noticed in both cell lines.

### 3.3 Influence of EGFR and MET level on invadopodia formation

As a result of our observation that EGFR and MET may regulate the invasion of primary and metastatic melanoma, we decided to put our attention to the invadopodia. They are actin‐rich protrusions crucial for cell movement through the ECM.^3^ Previously, we demonstrated, that tested A375 and WM9 cells are able to form these structures.^6^ Therefore, to evaluate the influence of differential expression level of EGFR and MET receptors on invadopodia formation, cortactin (a marker of these protrusions) and filamentous actin (F‐actin) were stained using immunocytochemistry (Figure 3B). Invadopodia were visible as dots in the cell nuclei proximity, where F‐actin and cortactin colocalized (which is indicated by white arrows in merge pictures, Figure 3B). Analysis of fluorescently labelled proteins showed increased number of invadopodia in A375 cells overexpressing EGFR and a contrary results were detected in WM9 shEGFR cells (Figure 3C). Moreover, decreased expression of MET also led to lowered number of invadopodia in examined cells.

### 3.4 Impact of EGFR and MET signalling on proteolytic activity of examined melanoma cells

**Figure 4:**
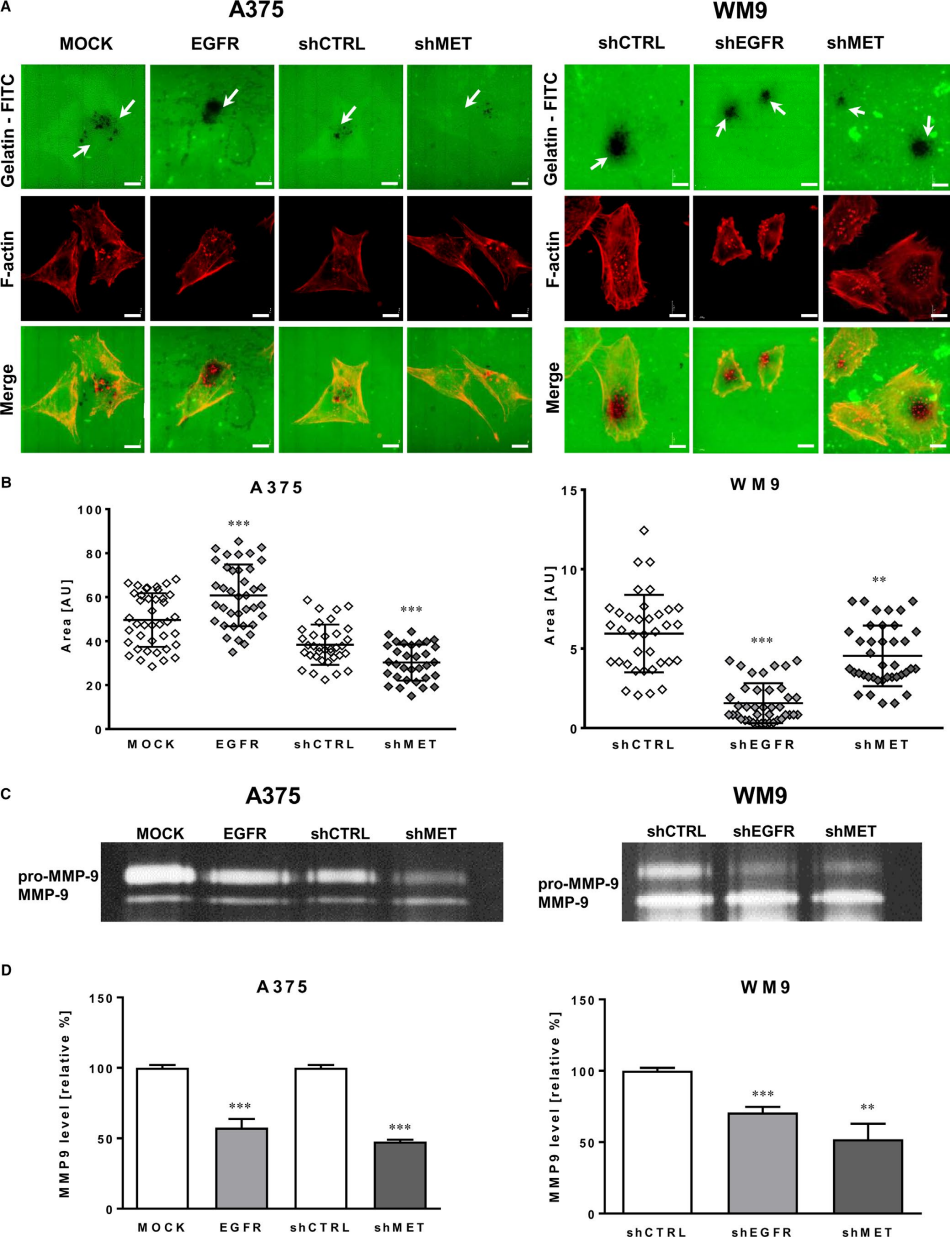
Proteolytic activity of melanoma cells with altered EGFR and MET expression. A, Representative pictures of proteolytic activity of A375 (MOCK, EGFR, shCTRL and shMET) and WM9 (shCTRL, shEGFR and shMET) cells (F‐actin visible in red) detected using FITC‐conjugated gelatin (green). Gelatin degradation indicated with white arrows is visualized as the dark areas on the fluorescently labelled gelatin background. Scale bar—10 µm. B, Quantification of digestion area calculated using ImageJ software from at least 40 cells from three independent experiments. Results are presented as the mean ± SD. C, MMP‐9 activity in concentrated conditioned media tested by gelatin zymography with (D) densitometric analysis. Results are expressed as the mean ± SD of three independent experiments. (**) *P* ≤ .01, (***) *P* ≤ .001

The main role of invadopodia is secretion of proteases digesting elements of the ECM, what enables cancer cells to invade through surrounding microenvironment and form metastasis.^4^, ^5^ Therefore, to estimate the proteolytic activity of tested cells, the gelatin‐FITC degradation assay was performed. In this test, sites of gelatin digestion appeared as black spots present on a fluorescently labelled background (white arrows, Figure 4A). Obtained data confirmed that all tested cells were able to digest gelatin mainly because of the activity of invadopodia. Next, the digested area corresponding to the proteolytic activity of cells was quantified (Figure 4B). The area was increased in A375 cells with up‐regulated EGFR expression level and lowered in WM9 shEGFR cells in comparison with control cells. Cells with silenced MET (A375 shMET and WM9 shMET) also presented decreased proteolytic activity and digested lower area in comparison with appropriate controls.

Moreover, we performed gelatin zymography, which is another way to test proteolytic activity of cells. We noticed that activity of MMP‐9 was lowered in cells with down‐regulated EGFR and MET protein level (Figure 4C), what was also confirmed by densytometric measurements (Figure 4D). Surprisingly, similar effect on MMP‐9 activity was induced by the overexpression of EGFR in A375 cells. We suppose that the level of other protease(s) present in these cells is elevated, since the surface of gelatin digestion is higher in EGFR overexpressing than in the control cells.

## 4 DISCUSSION

Recent studies consider receptor tyrosine kinases (RTKs) as the new potential molecular targets for melanoma treatment. In our studies, we focused on two of them—EGFR and MET receptors. Alterations in *EGFR* gene copy number in primary cutaneous malignant melanomas were associated with poor prognosis,^10^ while overexpression of EGFR was often detected in advanced stage of melanoma.^8^ MET was also demonstrated to be connected with malignant skin cancer development and the level of its expression seems to be related to the stage of malignancy in melanoma.^11^, ^22^ Moreover, based on our previous analysis we demonstrated that transcripts of both receptors are present in tumour tissue samples from patients suffering from melanoma (results for 114 primary and 155 metastatic melanoma samples from public database GEO).^15^ Similar results were obtained in the melanoma tumour samples collected and analysed by our group (data not shown). Therefore, both of these receptors emerge as promising therapeutic targets.

Signal transduction activated by EGFR has an important role in cell motility in various types of cancer.^23^, ^24^, ^25^ The crosstalk between EGFR and G‐protein–coupled receptors modulates Rho GTPases activity and may contribute to the cell migration.^26^, ^27^ In cancer cells, various mechanisms may lead to permanent activation of EGFR, that is overexpression of ligands and receptors, *EGFR* gene amplification or activating mutations. MET also regulates tumour cell migration, invasion and metastasis.^25^, ^28^ Signalling molecules activated by MET promote tumour metastasis by changing the expression of proteins involved in cytoskeletal rearrangements (cadherins, Arp2/3, N‐WASP) and cell adhesion (paxillin, integrins and focal adhesion kinase).^29^, ^30^, ^31^

Majority of the studies carried out on cancer cells focused only on verification how chemical inhibition of EGFR or MET activity affects cell viability^32^, ^33^ or tumour growth,^34^ what led to conclusions concerning involvement of these receptors in the regulation of tumour development. However, it is only part of the story since metastasis is the main cause of mortality among patients suffering from melanoma. Previously, we demonstrated that EGF and HGF stimulated invasiveness of melanoma cells.^6^ In this work, we tested two melanoma cell lines together with generated variants of them with stably modified expression of EGFR and MET, what allowed us to analyse the direct involvement of protein level of these receptors on the regulation of invasiveness of melanoma cells. To analyse it thoroughly, we investigated melanoma cell motility using several different assays both in 2D, reflecting the migration on the surface of basement membrane, and in 3D conditions, imitating invasion through the tissues. Our results indicate that both directed and spontaneous migrations (2D conditions) of melanoma cells are regulated by the EGFR and MET signalling. Analogous data were acquired in 3D conditions, where cells invaded through the layer of the Matrigel. Similarly, Lee and coworkers showed that ME22S (a novel EGFR/MET bispecific antibody) significantly inhibited HGF‐stimulated migration and invasion of laryngeal carcinoma cells.^35^ Moreover, it was shown that down‐regulation of EGFR caused by MiR‐615 and MiR‐7, led to decreased migration, and invasion of human glioblastoma and ovarian cancer cells, respectively.^36^, ^37^ Analogous effect was observed following MET silencing or its down‐regulation in many cancers like ovarian cancer, breast cancer, hepatocellular cancer or gastric cancer.^38^, ^39^ Corso et al^40^ also indicated that MET silencing in already established metastases led to their almost complete regression. Small molecule inhibitors (like crizotinib or foretinib) which block the activity of MET were also able to limit HGF‐stimulated melanoma cell migration^30^; however, these compounds may block activity of several kinases. Knockdown of MET, as well as selective inhibitors of EGFR, decreased proliferation of high MET‐expressing uveal melanoma cells. Moreover, uveal melanoma cell lines representing high expression of MET/EGFR possessed higher migration potential.^41^

Cell migration is the multi‐step process, where formation of actin‐rich protrusions is needed.^42^ We have previously shown that EGF and HGF stimulate invadopodia formation, and extracellular matrix degradation, what correlates with higher invasive abilities of melanoma cells.^6^ Interestingly, both EGFR and MET signalling also regulate invadopodia formation in breast cancer cells.^13^, ^14^ EGFR and MET inhibitors induced changes in actin cytoskeleton organization of oral squamous cell carcinoma cells. Furthermore, MET inhibitor reduced filopodia and lamellipodia formation, thus decreasing migration of these cells.^43^ Miekus et al^44^ also observed in MET‐deficient cervical carcinoma cells, that F‐actin was located under the cell membrane and did not form regular stress fibres which were present in control cells. Additionally, silencing of MET in cholangiocarcinoma cells led to the disappearance of actin‐rich protrusions induced by HGF.^45^ This is in line with our results, which indicate that the expression level of EGFR and MET correlates with number of invadopodia formed by melanoma cells. Therefore, EGFR and MET signalling may regulate cell migratory abilities by affecting their protrusive activity.

Secretion of proteases able to digest elements of the ECM enables cancer cells to invade through surrounding microenvironment and form metastasis.^4^, ^5^ MMP‐9 and MMP‐2 that induce degradation of the components of the extracellular matrix are particularly involved in favouring tumour cell infiltration and spreading.^46^, ^47^, ^48^ EGFR stimulation was demonstrated to promote squamous carcinoma cell migration and invasion *via* induction of EMT‐like phenotype switch and MMP‐9‐mediated degradation of E‐cadherin.^49^ Therefore, we also analysed proteolytic activity of generated variants of melanoma cells. We noticed that protein level of EGFR correlates with the ability to digest fluorescently labelled gelatin by melanoma cells. Further analysis revealed that activity of MMP‐9 was lowered in cells with down‐regulated EGFR. This is in line with observation of Zuo et al^49^ who showed that pharmacologic inhibition of EGFR activity reduced the production of MMP‐9, as well as squamous carcinoma cell migration and invasion. Zhen et al^50^ also indicated that knockdown of EGFR reduced cell invasion of gastric cancer and led to decreased expression of MMP‐9. Interestingly, reduced activity of MMP‐9 was induced by the overexpression of EGFR in A375 cells. Moreover, we did not notice changes in MMP‐2 activity in analysed variants of melanoma cell lines (data not shown). Matrix metalloproteinases and their tissue inhibitors play a crucial role in metastasis formation. Melanoma cells may express a several of matrix metalloproteinase family members (MMP‐1, MMP‐2, MMP‐7, MMP‐9, MMP‐13 and MT1‐MMP), as well as their tissue inhibitors (TIMP‐1, TIMP‐2 and TIMP‐3).^47^, ^48^ Therefore, it is possible that overexpression of EGFR led to up‐regulation of other type of MMP, what in consequence is balanced by decreased activity of MMP‐9; however, this hypothesis needs further studies. It was also found that MET signalling is essential for dendritic cell migration through the extracellular matrix, since both MMP‐2 activity and MMP‐9 activity were regulated by this receptor.^51^ Our results indicate that expression of MET receptor is also crucial for the proteolytic activity of melanoma cells—decreased digestion of fluorescently labelled gelatin and MMP‐9 activity were observed in melanoma cells with diminished level of this protein. Similar effect was observed by Sun et al^52^ who showed that MiR‐329 caused down‐regulation of MET expression what led to decreased mRNA level of MMP‐7 and MMP‐9 and thus reduced cellular migration and invasiveness of lung cancer cells.

In summary, our research presents the direct effect of EGFR and MET receptors protein level on the invasive abilities of melanoma cells. Obtained data indicate that both EGFR and MET signalling is strictly connected with migration and invasion abilities of melanoma cells, mostly because of the regulation of their proteolytic activity and the ability to form invadopodia. Therefore, these receptors seem to be good targets for anti‐melanoma therapy, which aim will be the reduction of metastasis.

## CONFLICT OF INTEREST

The authors confirm that there are no conflicts of interest.

## AUTHOR CONTRIBUTIONS

K.PG. and DN were involved in conceptualization and validation; K.PG. and ED performed formal analysis; K.PG., AS, ED, MP, MZ, RM and DN participated in investigation and writing‐review and editing; K.PG. and AS were involved in project administration; K.PG. wrote original draft; DN was involved in funding acquisition and supervision.

## Data Availability

The data that support the findings of this study are available from the corresponding author upon reasonable request.
